# Cannabis Use and the Risk of Cardiovascular Diseases: A Mendelian Randomization Study

**DOI:** 10.3389/fcvm.2021.676850

**Published:** 2021-08-02

**Authors:** Jianqiang Zhao, Heng Chen, Chengui Zhuo, Shudong Xia

**Affiliations:** ^1^Department of Cardiology, The Fourth Affiliated Hospital of Zhejiang University School of Medicine, Yiwu, China; ^2^Department of Cardiology, The First Affiliated Hospital of Zhejiang University School of Medicine, Hangzhou, China; ^3^Department of Cardiology, Taizhou Central Hospital, Taizhou University Hospital, Taizhou, China

**Keywords:** cannabis use, cardiovascular disease, Mendelian randomization, causation, genome-wide association studies

## Abstract

Several observational studies have shown that cannabis use has negative effects on the cardiovascular system, but the causality of this relationship has not been confirmed. The aim of the current study was to estimate the effects of genetically determined cannabis use on risk of cardiovascular diseases. Ten single-nucleotide polymorphisms related to cannabis use were employed as instruments to estimate the association between genetically determined cannabis use and risk of cardiovascular diseases using a two-sample Mendelian randomization (MR) method. Summary statistics data on exposure and outcomes were obtained from different genome-wide association meta-analysis studies. The results of this MR analysis showed no causal effects of cannabis use on the risk of several common cardiovascular diseases, including coronary artery disease, myocardial infarction, stroke and ischemic stroke subtypes, atrial fibrillation (AF), and heart failure. Various sensitivity analyses yielded similar results, and no heterogeneity and directional pleiotropy were observed. After adjusting for tobacco use and body mass index, multivariable MR analysis suggested a causal effect of cannabis use on small vessel stroke (SVS) [odds ratio (OR) 1.17; 95% CI 1.02–1.35; *p* = 0.03] and AF (OR 1.06; 95% CI 1.01–1.10; *p* = 0.01), respectively. This two-sample MR study did not demonstrate a causal effect of genetic predisposition to cannabis use on several common cardiovascular outcomes. After adjusting for tobacco use and body mass index, the multivariable MR analysis suggested a detrimental effect of cannabis use on the risk of SVS and AF, respectively.

## Introduction

In the past 20 years, there has been a rapid increase in cannabis use with the legalization of marijuana in some countries and regions. Global numbers of cannabis users reached an estimate of 188 million in 2017, which is roughly 3.8% of the global population aged 15–64 years (1). As cannabis is the most widely misused illicit drug, it is important to understand the impact of cannabis (or marijuana) on public health.

Many recent studies have shown that cannabis use has beneficial and detrimental effects on the cardiovascular system. Several observational studies have suggested that cannabis use is associated with cardiovascular diseases (CVDs), including coronary artery disease (CAD), myocardial infarction (MI), stroke, atrial fibrillation (AF), and heart failure (HF). In a retrospective cohort analysis by Chami et al. (2) that identified 292,770 patients with a history of cannabis abuse and 10,542,348 age- and sex-matched controls, cannabis abuse was significantly associated with incident MI [adjusted odds ratio (OR), 1.72; 95% CI 1.67–1.77; *p* < 0.0001]. Previous studies found that cannabis use independently predicted the risk of acute ischemic stroke among younger adults (adjusted OR, 1.17; 95% CI 1.15–1.20; *p* < 0.0001) (3) and HF in 18- to 55-year-old individuals (OR, 1.1; 95% CI 1.03–1.18; *p* < 0.01) (4) compared to non-users using data of the Nationwide Inpatient Sample (NIS) database. Several case reports have described the occurrence of AF following cannabis consumption, suggesting that cannabis use could be a cause of AF (5–7).

However, in other observational studies and systematic reviews, there was no strong evidence to confirm the previous findings (8, 9). In a large community-based cohort study of 5,115 young adults, neither cumulative lifetime nor recent use of cannabis was associated with increased risk of cardiovascular events, stroke, or cardiovascular mortality at middle age with a mean follow-up of 26.9 years (10). In addition, in a longitudinal study of the youngest cohort of the Pittsburgh Youth Study (PYS), greater cannabis exposure was associated with relatively lower risk of cardiovascular risk factors (11). Thus, the association between cannabis use and CVDs remains controversial.

In the last recent years, the Mendelian randomization (MR) approach has been widely used in assessing the causal effect of clinical factors on diseases (12). Based on the summarized data of genome-wide association meta-analysis studies (GWAS), the MR approach analyzes the causality between exposures and outcomes using genetic variants, usually single-nucleotide polymorphisms (SNPs). Random assignment of individual's genetic variants at conception are employed as instrumental variables. Thereby, MR analysis largely overcomes the limitations of environmental confounders (13, 14). Previous studies using MR have suggested a causal effect of cannabis use on the increased risk of development of schizophrenia (15, 16). However, the causal effect of cannabis use on CVDs has not been explored in a MR study. The aim of the current study was to estimate the effects of genetically determined cannabis use on risk of CVDs with a two-sample MR approach.

## Methods

The information on all the datasets used in this study is shown in Table 1. Ethical approval and participants' consent were obtained for all analyses.

**Table 1:** Details of studies and datasets used for analyses.

**Phenotype**	**Consortium**	**Ethnicity**	**Sample size**	**Cases**	**Use in this MR**	**References**
Cannabis Use	International Cannabis Consortium	European	32,330	14,387	Exposure	Stringer et al. (17)
Coronary heart disease	CARDIoGRAMplusC4D	77% European	184,305	60,801	Outcome	Nikpay et al. (18)
Myocardial infarction				42,560		
Any stroke	MEGASTROKE	Mixed	514,791	67,162	Outcome	Malik et al. (19)
Any ischemic stroke				60,341		
Large artery stroke				6,688		
Cardioembolic stroke				9,006		
Small vessel stroke				11,710		
Atrial fibrillation	AFGen, HUNT, MGI, deCODE, and UK Biobank	European	1,030,836	60,620	Outcome	Nielsen et al. (20)
Heart failure	HERMES	European	977,323	47,309	Outcome	Shah et al. (21)
Tobacco use	GSCAN	European	/	1,232,091	Mediator	Liu et al. (22)
Body mass index	GERA	European	100,418	/	Mediator	Hoffmann et al. (23)

*CARDIoGRAM-plusC4D, Coronary Artery Disease Genome-Wide Replication and Meta-analysis plus the Coronary Artery Disease; AFgen, Atrial Fibrillation Genetics; HUNT, Nord-Trøndelag Health Study; MGI, Michigan Genomics Initiative; deCOCE, Collaborative Analysis of Diagnostic Criteria in Europe; HERMES, Heart Failure Molecular Epidemiology for Therapeutic Targets, GSCAN, GWAS & Sequencing Consortium of Alcohol and Nicotine; GERA, Genetic Epidemiology Research on Adult Health and Aging*.

### Data Sources

The 10 leading SNPs from the International Cannabis Consortium (ICC) GWAS (17), which explain 13–20% of the observed phenotypic variation of cannabis use (ever/never used cannabis during lifetime) in 32,330 participants of European ancestry, were selected as instruments for the MR analyses. Although, none of the SNPs reached the conventional genome-wide significance threshold (*p* < 5 × 10^−8^), estimates were directionally consistent across the vast majority of contributing studies. Therefore, these SNPs are considered effective instruments for MR analysis (24). In addition, the *F*-statistic was calculated to evaluate the strength of each instrument. A threshold of *F*-statistic >10 suggests that the genetic variants have strong estimated effects in an MR analysis (25). The corresponding linkage disequilibrium was tested on the LD-link website (https://ldlink.nci.nih.gov/, European; r^2^ < 0.1) to ensure the selected SNPs were independent (26). Furthermore, genome-wide traits that were significantly (*p* <5 × 10^−8^) associated with these SNPs were searched using the PhenoScanner (http://www.phenoscanner.medschl.cam.ac.uk/) to evaluate the confounding factors in the potential association between cannabis use and increased risk of CVDs (27).

Genetic association estimates for CAD and MI were extracted from the Coronary Artery Disease Genome-Wide Replication and Meta-analysis plus the Coronary Artery Disease (CARDIoGRAM-plusC4D) 1,000 genomes-based GWAS meta-analysis of 48 studies, which contained 60,801 CAD cases (≈70% had MI) and 123,504 controls, with 77% of participants being of European ancestry (18). For stroke and ischemic stroke subtypes, data were derived from a multi-ancestry GWAS of 29 studies in the MEGASTROKE consortium including 67,162 cases of any stroke (AS), 60,341 cases of any ischemic stroke (AIS), 6,688 cases of large artery stroke (LAS), 9,006 cases of cardioembolic stroke (CES), and 11,710 cases of small vessel stroke (SVS) (19). Genetic association data for AF were obtained from the meta-analysis of GWAS for AF by the Atrial Fibrillation Genetics (AFGen) Consortium, the Nord-Trøndelag Health Study (HUNT), the Michigan Genomics Initiative (MGI), DiscovEHR, Collaborative Analysis of Diagnostic Criteria in Europe study (deCODE), and UK Biobank, which included 1,030,836 individuals of European ancestry (60,620 with AF and 970,216 controls) (20). For the effects of SNPs on HF, summary statistics from the Heart Failure Molecular Epidemiology for Therapeutic Targets (HERMES) Consortium was used, which included 47,309 cases and 930,014 control subjects of European ancestry from 26 studies (21).

### Statistical Analysis

A two-sample MR method was used in the current study. The Wald estimator to derive MR estimates of the effect of cannabis use on CVDs was used, which is the ratio of SNP-outcome genetic effect over SNP-exposure genetic effect. The Delta method was used to account for possible measurement errors in both the exposure and outcome association estimates (28, 29). The fixed-effects inverse variance-weighted (IVW) method was used to derive the final effect estimate for the main analyses. Weighted median and MR-Egger regression methods were applied in sensitivity analyses (30). Directional pleiotropy was assessed by estimating the deviation of MR-Egger intercepts (31). Heterogeneity was measured by *I*^2^ and Cochran Q-derived P. Furthermore, the leave-one-out analysis was used to determine any pleiotropy influenced by a single SNP. In addition, multivariable MR was performed to test the effect of tobacco use and body mass index (BMI) on causal estimates. The effect of tobacco use was explored using data from GWAS & Sequencing Consortium of Alcohol and Nicotine (GSCAN), including 1,232,091 individuals who had smoked regularly (22), and the effect of BMI was explored using data from the single large multi-ethnic Genetic Epidemiology Research on Adult Health and Aging (GERA) cohort, containing average BMI measurements of 100,418 adults (23). Statistical power was calculated using the mRnd power calculator (available at http://cnsgenomics.com/shiny/mRnd/) (32). As indicated, for >80% statistical power, the OR should be <0.962 or >1.038 for the cannabis use–CVD relationship, <0.957 or >1.043 for the cannabis use–MI relationship, <0.968 or >1.032 for the cannabis use–AS relationship, <0.966 or >1.034 for the cannabis use–AIS relationship, <0.905 or >1.095 for the cannabis use–LAS relationship, <0.916 or >1.084 for the cannabis use–CES relationship, <0.928 or >1.072 for the cannabis use–SVS relationship, <0.968 or >1.032 for the cannabis use–AF relationship, and <0.964 or >1.036 for the cannabis use–HF relationship (Supplementary Table 1). All statistical analyses were performed using R software (version 3.6.1) with the Mendelian Randomization package. A Bonferroni-corrected level of significance of <0.006 (0.05/9 outcomes) was considered to indicate statistical significance. *p*-values between 0.008 and 0.05 were regarded as suggested associations.

## Results

The details of the characteristics of the SNPs for cannabis use are shown in Table 2. All 10 SNPs had an *F*-statistic above the threshold of 10, which suggested that they strongly predicted cannabis use in the MR analysis. The genetic associations between the selected SNPs and all outcomes and tobacco use are presented in Supplementary Tables 2–6 (Supplemental data). The SNP rs4471463 was associated with the smoking status “ever smoked,” rs35053471 was associated with lymphocyte count, and rs73067624 was associated with age-related macular degeneration (PhenoScanner, Supplementary Table 7, Supplemental data).

**Table 2:** Characteristics of SNPs for cannabis use from GWAS meta-analysis.

**SNP**	**Chromosome position**	**EA**	**EAF**	***R*^**2**^ (%)**	***F***	**Beta**	**SE**	***p*-value**
rs35053471	3:47124761:T:A	T	0.62	0.38	12.3	0.090	0.022	2.7 × 10^−6^
rs12518098	5:60864467:C:G	C	0.68	0.35	11.4	0.090	0.023	3.0 × 10^−6^
rs4471463	11:112983595:C:T	C	0.45	0.50	16.2	0.100	0.021	1.5 × 10^−6^
rs4984460	15:96424399:T:G	G	0.25	0.45	14.7	0.110	0.023	4.6 × 10^−7^
rs7675351	4:141218757:A:C	C	0.14	0.41	13.3	0.130	0.033	1.4 × 10^−6^
rs73067624	1:196333461:T:C	C	0.10	0.46	14.9	0.160	0.041	3.1 × 10^−6^
rs2099149	12:30479358:T:G	G	0.19	0.89	29.0	0.170	0.034	9.8 × 10^−7^
rs2033867	2:175188281:G:A	A	0.06	0.60	19.5	0.230	0.050	2.6 × 10^−6^
rs58691539	2:52526771:T:G	G	0.09	1.38	45.2	0.290	0.062	2.1 × 10^−6^
rs7107977	11:915764:A:G	A	0.60	4.04	136.1	0.290	0.064	1.9 × 10^−6^

*SNP, single-nucleotide polymorphism; EA, effect allele; EAF, effect allele frequency; R^2^, percentage of the variation explained by the SNP; F, F statistic; Beta, the per-allele effect on cannabis use; SE, standard error of Beta; p-value is for the genetic association.*

*R^2^ = 2 × minor allele frequency × (1 – minor allele frequency) × (β/SD)^2^.*

*F = R^2^(n – 2)/k(1 – R^2^), n stands for the sample size (n = 32,330) and k stands for the number of instruments*.

**Figure 1:**
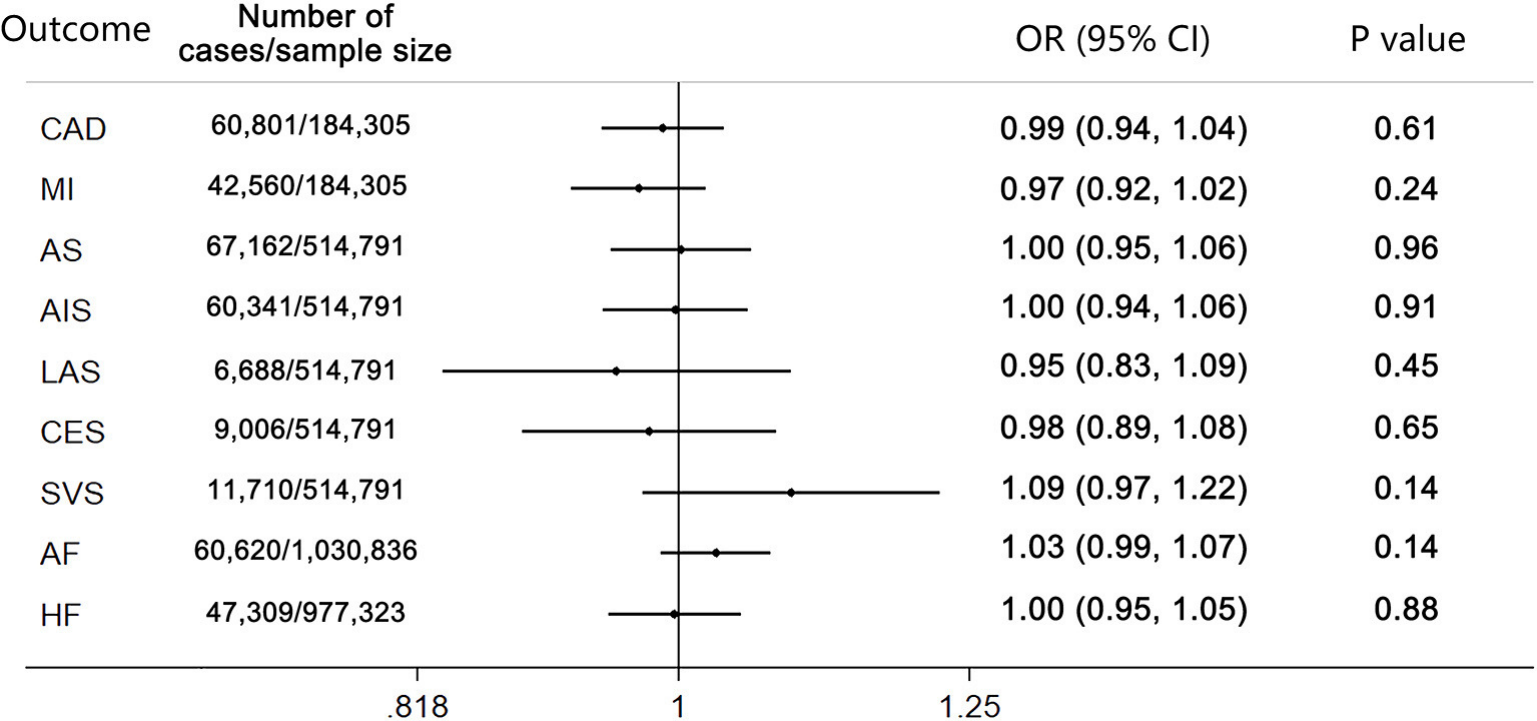
Mendelian randomization analysis estimates for cannabis use on risk of cardiovascular diseases. *p*-value for the association between the SNPs and outcomes. CAD, coronary artery disease; MI, myocardial infarction; AS, any stroke; AIS, any ischemic stroke; LAS, large artery stroke; CES, cardioembolic stroke; SVS, small vessel stroke.

**Figure 2:**
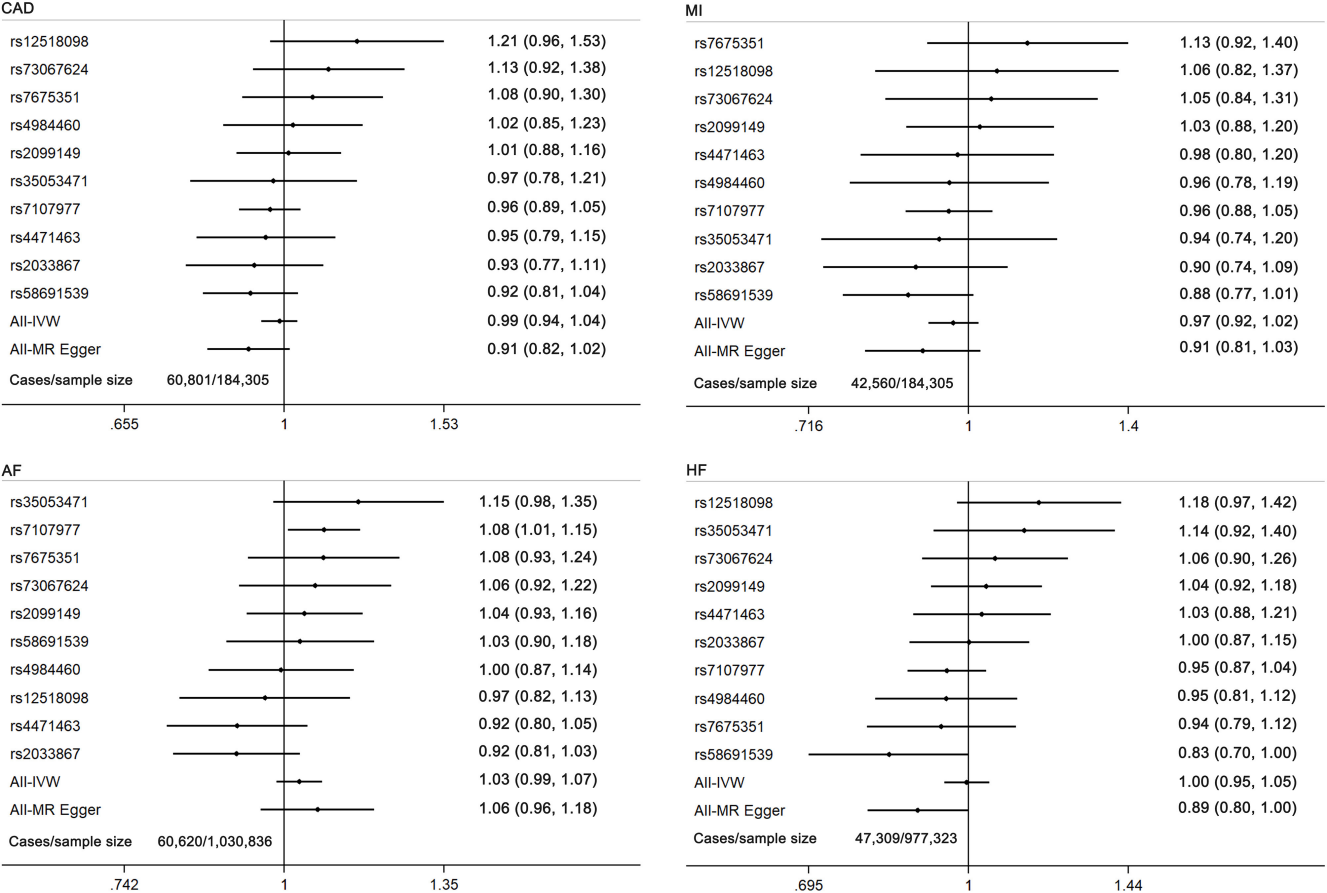
Forest plot of MR estimates for the causal effect of each cannabis use marker on coronary artery disease, myocardial infarction, atrial fibrillation, and heart failure. CAD, coronary artery disease; MI, myocardial infarction; AF, atrial fibrillation; HF, heart failure.

**Figure 3:**
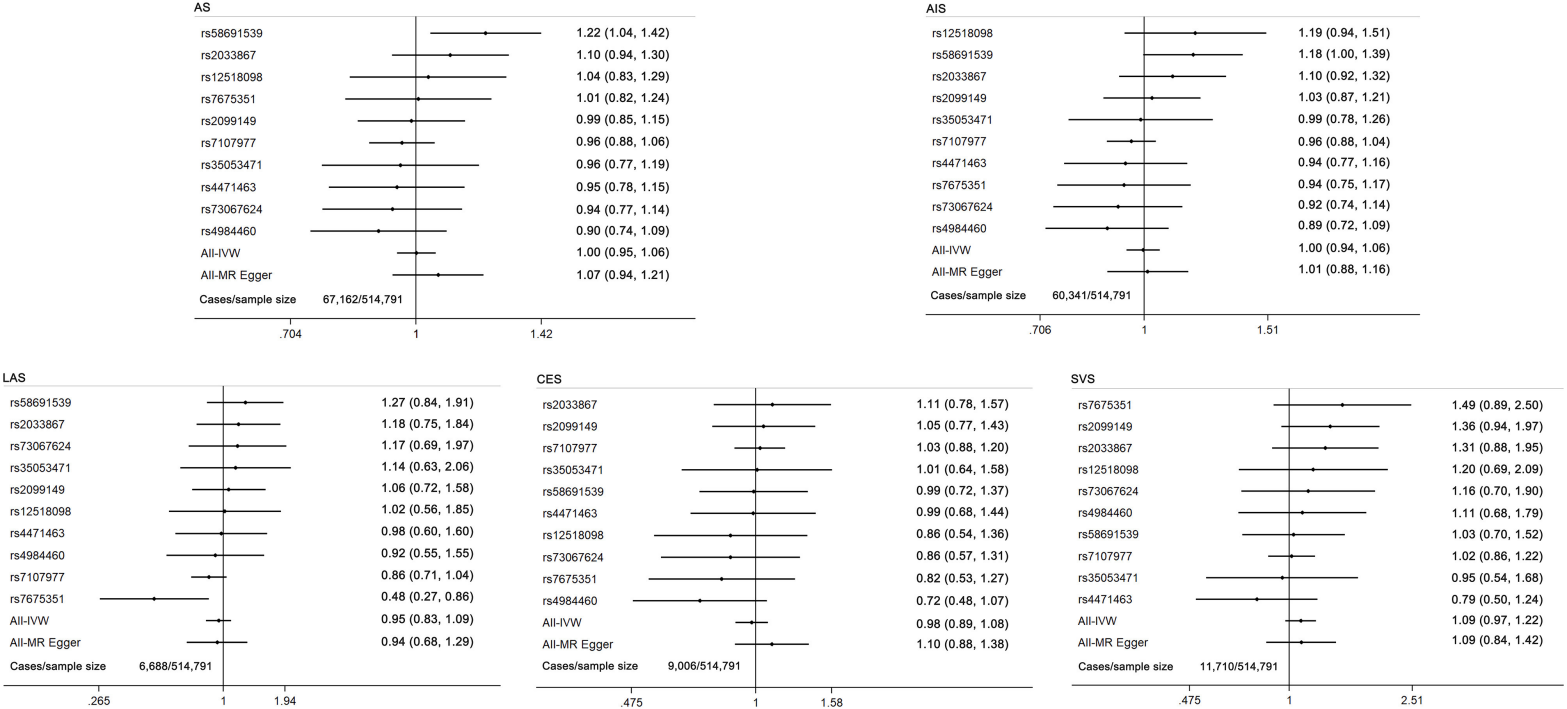
Forest plot of MR estimates for the causal effect of each cannabis use marker on any stroke and ischemic stroke subtypes. AS, any stroke; AIS, any ischemic stroke; LAS, large artery stroke; CES, cardioembolic stroke; SVS, small vessel stroke.

The results of the MR analysis estimates for the effect of cannabis use on risk of CVDs are shown in Figure 1. The IVW analysis results per 1-log unit increase in ever use of cannabis on CAD and MI were OR 0.99 (95% CI 0.94–1.04, *p* = 0.61) and OR 0.97 (95% CI 0.92–1.02, *p* = 0.24), respectively. Also, no causal association was observed between cannabis use and risk of any stroke or ischemic stroke subtypes (AS: OR 1.00; 95% CI 0.95–1.06; *p* = 0.96; AIS: OR 1.00; 95% CI 0.94–1.06; *p* = 0.91; LAS: OR 0.95; 95% CI 0.83–1.09; *p* = 0.45; CES: OR 0.98; 95% CI 0.89–1.08; *p* = 0.65; SVS: OR 1.09; 95% CI 0.97–1.22; *p* = 0.14). Moreover, there was no causal effect of cannabis use on the risk of AF (OR 1.03; 95% CI 0.99–1.07; *p* = 0.14) or HF (OR 1.00; 95% CI 0.95–1.05; *p* = 0.88). Forest plots of a single SNP effect on outcomes did not reveal associations between cannabis use and CVDs (Figures 2, 3).

In sensitivity analyses, the weighted median and MR-Egger regression methods yielded consistent results (Supplementary Table 8). However, cannabis use was associated with HF in the MR-Egger analysis (OR 0.82; 95% CI 0.80–1.00; *p* = 0.05) and directional pleiotropy test (*p* = 0.036), which suggested an association between cannabis use and HF after correcting for the bias of directional pleiotropy. There was no evidence of directional pleiotropy or heterogeneity in the IVW analysis for other outcomes (Supplementary Table 9). The leave-one-out analysis did not find any pleiotropy influenced by a single SNP and confirmed the lack of associations (Supplementary Tables 10–12).

The multivariable MR analyses investigated the robustness of the association of genetically predicted cannabis use with CVDs. The results are consistent in the adjusted model (Table 3). However, after adjusting for tobacco use, a causal effect of cannabis use on SVS was suggested (OR 1.17; 95% CI 1.02–1.35; *p* = 0.03). After adjusting for BMI, the analyses suggested a causal effect of cannabis use on AF (OR 1.06; 95% CI 1.01–1.10; *p* = 0.01).

**Table 3:** Multivariable MR associations of cannabis use with cardiovascular diseases adjusted for tobacco use and BMI.

**Outcome**	**Method**	**OR (95% CI)**	***p*-value**
CAD	IVW adjusted for Tobacco	1.06 (0.98–1.15)	0.16
	IVW adjusted for BMI	0.99 (0.94–1.05)	0.77
MI	IVW adjusted for Tobacco	1.02 (0.93–1.12)	0.65
	IVW adjusted for BMI	0.97 (0.92–1.03)	0.36
AS	IVW adjusted for Tobacco	1.00 (0.92–1.08)	0.93
	IVW adjusted for BMI	1.00 (0.94–1.06)	0.90
AIS	IVW adjusted for Tobacco	1.00 (0.92–1.10)	0.92
	IVW adjusted for BMI	0.99 (0.93–1.05)	0.75
LAS	IVW adjusted for Tobacco	1.01 (0.84–1.20)	0.93
	IVW adjusted for BMI	0.95 (0.85–1.06)	0.36
CES	IVW adjusted for Tobacco	0.92 (0.79–1.08)	0.30
	IVW adjusted for BMI	0.97 (0.88–1.07)	0.55
SVS	IVW adjusted for Tobacco	1.17 (1.02–1.35)	0.03
	IVW adjusted for BMI	1.07 (0.98–1.16)	0.15
AF	IVW adjusted for Tobacco	1.03 (0.97–1.10)	0.32
	IVW adjusted for BMI	1.06 (1.01–1.10)	0.01
HF	IVW adjusted for Tobacco	1.03 (0.96–1.10)	0.45
	IVW adjusted for BMI	0.99 (0.93–1.05)	0.74

*CAD, coronary artery disease; MI, myocardial infarction; AS, any stroke; AIS, any ischemic stroke; LAS, large artery stroke; CES, cardioembolic stroke; SVS, small vessel stroke; BMI, body mass index; p-value is for the genetic association*.

## Discussion

Results of the current MR study did not provide evidence that genetically determined cannabis use had a detrimental effect on CVDs. Sensitivity analyses and multivariable MR analyses confirmed this conclusion.

With the increased prevalence of both medicinal and recreational use of cannabis, several observational epidemiological studies have demonstrated the association between cannabis use and risk of CVDs. In a retrospective study including 2,451,933 acute MI patients, multivariable analysis showed that cannabis use raised the risk of acute MI by 3–8% compared with no history of cannabis use (33). According to a meta-analysis evaluating risk factors of MI, smoking cannabis was identified as the third ranking trigger for MI (34). Similarly, Kalla et al. (4) concluded that cannabis use was an independent predictor of both HF and cerebrovascular accident after analyzing data from the NIS database. However, other evidence pointed to the opposite conclusion that cannabis use was not an independent risk factor for CVDs. In a prospective cohort study, after multivariate regression adjustment, the association between cannabis use and cardiovascular risk factors was no longer significant, which was mainly caused by adjusting for the confounder of alcohol use (35). In addition, a population case–cohort study found no evident association between cannabis use and stroke when adjusted for tobacco use (36). Assessing the effects of cannabis use on the cardiovascular system is complicated though, partly due to pharmacology, onset of age, drug dose, user patterns, and type of formulations of cannabis (37). Therefore, this study was timely to draw attention to the evaluation of the causal effect of cannabis use on CVDs.

The potential mechanism of effects of cannabis use on increased risk of CVDs may be mediated by the opposing effects of cannabinoid on the cardiovascular system, through the cannabinoid-1 (CB_1_) and cannabinoid-2 (CB_2_) receptors (38). CB_1_ receptors accelerate endothelial cell growth and proliferation (39) and play a role in the formation of oxidized low-density lipoprotein and the induction of an inflammatory response (40). Conversely, CB_2_ receptors may reduce progression of atherosclerosis by reducing levels of reactive oxygen species (41) and attenuation of pro-inflammatory processes (9). Inflammatory response and oxygen supply/demand mismatch caused by cannabis have also been linked to the development of CAD and MI, while pathophysiological mechanisms for arrhythmias and endothelial damage may be due to the hyperadrenergic state and oxidative stress (42).

In addition, cannabis use has been considered to be associated with unhealthy behaviors, such as sleep disorder (43), underweight status (44), tVobacco (45), and alcohol (46) use, and other illicit drug use, which all may lead to increased risk of cardiovascular events. A longitudinal study of 503 boys found that cannabis use was associated with lower BMI and reduced risk of other cardiometabolic risk factors (11). However, after adjusting for BMI, these associations were no longer apparent, which suggested that lower BMI in cannabis users might explain their lower levels of risk on CVDs. Likewise, our study showed a causal effect of cannabis use on AF in multivariable MR analyses after adjusting for BMI, suggesting that the mediation effect of BMI might mask the effect of cannabis use on CVDs. In addition, GWAS of cannabis use reported a strong genetic correlation between cannabis use and cigarette smoking (17). A previous MR study found that smoking is a risk factor for several CVD outcomes, including MI, HF, and large artery atherosclerosis (47). By analyzing data of 3,117 participants with artery calcium measurements, Auer et al. found that cannabis use was associated with subclinical atherosclerosis, but only among ever tobacco users (48). Interestingly, another study showed the opposite conclusion that cannabis use was associated with a significantly higher risk of several CVDs, particularly in non-tobacco users (49). Therefore, multivariable MR was performed to explore the effect of tobacco use on the potential association between cannabis use and CVDs. After adjusting for tobacco use, the analyses suggested a causal effect of cannabis use on increased risk of SVS.

In general, the association between cannabis use and CVDs remains controversial, because of the presence of confounding risk factors in observational studies. In this MR study, the association between genetically predicted cannabis use and risk of CVDs was investigated. Strengths of our study included the Mendelian randomization design, which could largely overcome the limitations of observational studies with environmental confounders and minimize reverse causation bias, thereby providing high-quality evidence. Second, the selected SNPs explained a relatively high proportion (13%) of the observed phenotypic variation of cannabis use. Another strength is the large sample size of each MR analysis and the strong estimated effects of each genetic variant (all *F*-statistic >10). Therefore, this study had high statistical power to assess the potential association of cannabis use with CVDs.

This study had several limitations. First, none of the selected SNPs reached the conventional genome-wide significance threshold. Nevertheless, estimates were directionally consistent across the vast majority of contributing studies and instrumental variables explained a relatively high proportion of cannabis use. Therefore, these SNPs can be considered as effective instruments for MR analysis. In addition, results of sensitivity analyses showed stability of the causal estimates. Second, due to the limitation on categories of data from the ICC GWAS, the risk of CVDs in relation to the onset of age, drug dose, use patterns, and type of cannabis could not be investigated. Third, the mechanism of the effect of cannabis use on risk of CVDs remains unknown, and there was no relationship between other environmental factors and CVDs. Finally, not all participants included in some GWAS were of European ancestry, and the variability of allele frequencies between populations may have an impact on the results. Lastly, the findings may not be generalizable to other populations.

## Conclusion

In conclusion, the current MR study suggested that genetic predisposition to cannabis use was not causally associated with CVDs. Conflicting findings from observational studies might have resulted from residual confounding. Large intervention studies are required to explore the effectiveness of stopping use of cannabis on reducing the incidence of CVDs.

## Data Availability Statement

The original contributions presented in the study are included in the article/Supplementary Material, further inquiries can be directed to the corresponding author/s.

## Author Contributions

JZ designed the study, contributed to the data analysis, and wrote the manuscript. CZ contributed to the data collection and data analysis. SX contributed to manuscript writing. HC contributed to manuscript writing and revision of the manuscript. All authors read and approved the final draft of the manuscript.

## Conflict of Interest

The authors declare that the research was conducted in the absence of any commercial or financial relationships that could be construed as a potential conflict of interest.

## Publisher's Note

All claims expressed in this article are solely those of the authors and do not necessarily represent those of their affiliated organizations, or those of the publisher, the editors and the reviewers. Any product that may be evaluated in this article, or claim that may be made by its manufacturer, is not guaranteed or endorsed by the publisher.

## Abbreviations

CVD: cardiovascular disease
GWAS: genome-wide association study
CAD: coronary artery disease
MI: myocardial infarction
AF: atrial fibrillation
HF: heart failure
MR: Mendelian randomization
SNP: single-nucleotide polymorphism
ICC: International Cannabis Consortium
CARDIoGRAM-plusC4D: Coronary Artery Disease Genome-Wide Replication and Meta-analysis plus the Coronary Artery Disease
AS: any stroke
AIS: any ischemic stroke
LAS: large artery stroke
CES: cardioembolic stroke
SVS: small vessel stroke
AFgen: Atrial Fibrillation Genetics
HUNT: Nord-Trøndelag Health Study
MGI: Michigan Genomics Initiative
deCOCE: Collaborative analysis of Diagnostic criteria in Europe
HERMES: Heart Failure Molecular Epidemiology for Therapeutic Targets
IVW: inverse variance-weighted
GSCAN: GWAS & Sequencing Consortium of Alcohol and Nicotine
BMI: body mass index
GERA: Genetic Epidemiology Research on Adult Health and Aging
OR: odds ratio
CI: confidence interval.
