# Supplementary material for: Cannabis Use and the Risk of Cardiovascular Diseases: A Mendelian Randomization Study
Source: Front Cardiovasc Med. 2021 Aug 2;8:676850. doi: 10.3389/fcvm.2021.676850 (PMC8366226; doi:10.3389/fcvm.2021.676850)
Supplement: Supplementary file 1 [file Data_Sheet_1.docx]

Supplementary Material

Table S1. Power for conventional Mendelian randomization analysis

| Outcome | Sample size | Proportion of cases | R^2^ of instruments | OR required for 80% power |
| --- | --- | --- | --- | --- |
| CAD | 184,305 | 0.33 | 0.13 | 0.962 (1.038) |
| MI | 184,305 | 0.23 | 0.13 | 0.957 (1.043) |
| AS | 514,791 | 0.13 | 0.13 | 0.968 (1.032) |
| AIS | 514,791 | 0.12 | 0.13 | 0.966 (1.034) |
| LAS | 514,791 | 0.013 | 0.13 | 0.905 (1.095) |
| CES | 514,791 | 0.017 | 0.13 | 0.916 (1.084) |
| SVS | 514,791 | 0.023 | 0.13 | 0.928 (1.072) |
| AF | 1,030,836 | 0.059 | 0.13 | 0.968 (1.032) |
| HF | 977,323 | 0.048 | 0.13 | 0.964 (1.036) |

R^2^, percentage of the variation explained by the SNP; CAD, coronary artery disease; MI, myocardial infarction; AS, any stroke; AIS, any ischemic stroke; LAS, large artery stroke; CES, cardioembolic stroke; SVS, small vessel stroke; AF, atrial fibrillation; HF, heart failure.

(available at http://cnsgenomics.com/shiny/mRnd/)

Table S2. The raw information of the selected SNPs with coronary artery disease and myocardial infarction from GWAS

| SNP | EA | Coronary artery disease | | Myocardial infarction | |
| --- | --- | --- | --- | --- | --- |
|  |  | β(SE) | P value | β(SE) | P value |
| rs35053471 | T | -0.0025  0.0102 | 0.807 | -0.0055  0.0113 | 0.629 |
| rs12518098 | C | 0.0174  0.0106 | 0.101 | 0.0053  0.0117 | 0.648 |
| rs4471463 | C | -0.0048  0.0094 | 0.608 | -0.0023  0.0104 | 0.825 |
| rs4984460 | G | 0.0025  0.0104 | 0.807 | -0.0044  0.0117 | 0.710 |
| rs7675351 | C | 0.0099  0.0124 | 0.427 | 0.0162  0.0140 | 0.247 |
| rs73067624 | C | 0.0190  0.0164 | 0.247 | 0.0077  0.0182 | 0.671 |
| rs2099149 | G | 0.0022  0.0121 | 0.858 | 0.0042  0.0135 | 0.756 |
| rs2033867 | A | -0.0180  0.0214 | 0.400 | -0.0255  0.0226 | 0.261 |
| rs58691539 | G | -0.0258  0.0186 | 0.165 | -0.0365  0.0202 | 0.070 |
| rs7107977 | A | -0.0108  0.0121 | 0.370 | -0.0118  0.0134 | 0.379 |

SNP, single-nucleotide polymorphism; Beta, the per allele effect on cannabis use; SE, standard error of Beta; P value is for the genetic association.

Table S3. The raw information of the selected SNPs with any stroke and ischemic stroke subtypes from GWAS

| SNP | EA | Any stroke | | Any ischemic stroke | | | Large artery stroke | | Cardioembolic stroke | | Small vessel stroke | |
| --- | --- | --- | --- | --- | --- | --- | --- | --- | --- | --- | --- | --- |
|  |  | β(SE) | P value | | β(SE) | P value | β(SE) | P value | β(SE) | P value | β(SE) | P value |
| rs35053471 | T | -0.0039  0.0101 | 0.698 | -0.0010  0.0111 | | 0.931 | 0.0119  0.0271 | 0.660 | 0.0008  0.0207 | 0.968 | -0.0046  0.0261 | 0.860 |
| rs12518098 | C | 0.0031  0.0101 | 0.762 | 0.0154  0.0109 | | 0.158 | 0.0017  0.0274 | 0.950 | -0.0135  0.0211 | 0.523 | 0.0162  0.0256 | 0.527 |
| rs4471463 | C | -0.0053  0.0097 | 0.583 | -0.0062  0.0105 | | 0.551 | -0.0021  0.025 | 0.933 | -0.0015  0.0192 | 0.937 | -0.0242  0.0232 | 0.296 |
| rs4984460 | G | -0.0115  0.0107 | 0.282 | -0.0136  0.0116 | | 0.239 | -0.0091  0.0293 | 0.755 | -0.0367  0.0226 | 0.105 | 0.011  0.0269 | 0.683 |
| rs7675351 | C | 0.0009  0.0136 | 0.948 | -0.0087  0.0148 | | 0.556 | -0.0958  0.0386 | 0.013 | -0.0263  0.0292 | 0.368 | 0.052  0.0342 | 0.128 |
| rs73067624 | C | -0.0105  0.0160 | 0.512 | -0.0140  0.0176 | | 0.427 | 0.0244  0.0427 | 0.569 | -0.0241  0.0341 | 0.480 | 0.023  0.0408 | 0.572 |
| rs2099149 | G | -0.0021  0.0131 | 0.873 | 0.0045  0.0141 | | 0.751 | 0.0103  0.0342 | 0.763 | 0.0083  0.0269 | 0.759 | 0.0525  0.0319 | 0.099 |
| rs2033867 | A | 0.0222  0.0192 | 0.246 | 0.0221  0.0210 | | 0.292 | 0.0372  0.0526 | 0.480 | 0.0231  0.0413 | 0.576 | 0.0621  0.0468 | 0.184 |
| rs58691539 | G | 0.0567  0.0230 | 0.014 | 0.0475  0.0247 | | 0.055 | 0.0684  0.0606 | 0.259 | -0.0034  0.048 | 0.944 | 0.0095  0.0567 | 0.867 |
| rs7107977 | A | -0.0111  0.0136 | 0.415 | -0.0123  0.0122 | | 0.314 | -0.0439  0.0283 | 0.122 | 0.0075  0.0225 | 0.740 | 0.0057  0.0259 | 0.825 |

SNP, single-nucleotide polymorphism; Beta, the per allele effect on cannabis use; SE, standard error of Beta; P value is for the genetic association.

Table S4. The raw information of the selected SNPs with atrial fibrillation from GWAS

| SNP | EA | Atrial fibrillation | |
| --- | --- | --- | --- |
|  |  | β(SE) | P value |
| rs35053471 | T | 0.0125  0.0073 | 0.087 |
| rs12518098 | C | -0.0032  0.0073 | 0.662 |
| rs4471463 | C | -0.0087  0.0067 | 0.195 |
| rs4984460 | G | -0.0006  0.0076 | 0.934 |
| rs7675351 | C | 0.0096  0.0094 | 0.309 |
| rs73067624 | C | 0.0093  0.0116 | 0.426 |
| rs2099149 | G | 0.0065  0.0094 | 0.490 |
| rs2033867 | A | -0.0203  0.0139 | 0.145 |
| rs58691539 | G | 0.0087  0.0204 | 0.670 |
| rs7107977 | A | 0.0217  0.0100 | 0.031 |

SNP, single-nucleotide polymorphism; Beta, the per allele effect on cannabis use; SE, standard error of Beta; P value is for the genetic association.

Table S5. The raw information of the selected SNPs with heart failure from GWAS

| SNP | EA | Heart failure | |
| --- | --- | --- | --- |
|  |  | β(SE) | P value |
| rs35053471 | T | 0.0115  0.0095 | 0.225 |
| rs12518098 | C | 0.0145  0.0086 | 0.091 |
| rs4471463 | C | 0.0031  0.0080 | 0.701 |
| rs4984460 | G | -0.0055  0.0091 | 0.548 |
| rs7675351 | C | -0.0080  0.0113 | 0.483 |
| rs73067624 | C | 0.0098  0.0136 | 0.471 |
| rs2099149 | G | 0.0070  0.0110 | 0.527 |
| rs2033867 | A | 0.0005  0.0161 | 0.976 |
| rs58691539 | G | -0.0525  0.0271 | 0.052 |
| rs7107977 | A | -0.0141  0.0133 | 0.291 |

SNP, single-nucleotide polymorphism; Beta, the per allele effect on cannabis use; SE, standard error of Beta; P value is for the genetic association.

Table S6. The raw information of the selected SNPs with tobacco use from GWAS

| SNP | EA | Tobacco use | |
| --- | --- | --- | --- |
|  |  | β(SE) | P value |
| rs35053471 | T | -0.0034  0.0019 | 0.030 |
| rs12518098 | C | 0.0005  0.0020 | 0.487 |
| rs4471463 | C | -0.0138  0.0018 | 1.94E-17 |
| rs4984460 | G | 0.0006  0.0021 | 0.624 |
| rs7675351 | C | 0.0003  0.0025 | 0.484 |
| rs73067624 | C | -0.0025  0.0032 | 0.329 |
| rs2099149 | G | -0.0047  0.0028 | 0.073 |
| rs2033867 | A | -0.0099  0.0037 | 0.005 |
| rs58691539 | G | \ | \ |
| rs7107977 | A | \ | \ |

SNP, single-nucleotide polymorphism; Beta, the per allele effect on cannabis use; SE, standard error of Beta; P value is for the genetic association.

Table S6. The raw information of the selected SNPs with BMI from GWAS

| SNP | EA | BMI | |
| --- | --- | --- | --- |
|  |  | β(SE) | P value |
| rs35053471 | T | 0.0022  0.0029 | 0.43 |
| rs12518098 | C | -0.0050  0.0030 | 0.10 |
| rs4471463 | C | -0.0187  0.0028 | 1.90E-11 |
| rs4984460 | G | -0.0032  0.0032 | 0.32 |
| rs7675351 | C | 0.0026  0.0041 | 0.53 |
| rs73067624 | C | -0.0010  0.0048 | 0.84 |
| rs2099149 | G | 0.0011  0.0031 | 0.72 |
| rs2033867 | A | -0.0121  0.0056 | 0.03 |
| rs58691539 | G | -0.0033  0.0049 | 0.50 |
| rs7107977 | A | -0.0039  0.0030 | 0.20 |

SNP, single-nucleotide polymorphism; Beta, the per allele effect on cannabis use; SE, standard error of Beta; P value is for the genetic association.

Table S7. Related traits of cannabis-associated SNPs.

| SNP | Chromosome: Position | Trait | Beta | P value |
| --- | --- | --- | --- | --- |
| rs35053471 | 3:47124761:T:A | Lymphocyte count | 0.0524 | 3.50E-44 |
| rs12518098 | 5:60864467:C:G | NA | NA | NA |
| rs4471463 | 11:112983595:C:T | Ever smoked | 0.0113 | 4.37E-21 |
| rs4984460 | 15:96424399:T:G | NA | NA | NA |
| rs7675351 | 4:141218757:A:C | NA | NA | NA |
| rs73067624 | 1:196333461:T:C | Age-related macular degeneration | NA | 5.13×10^-31^ |
| rs2099149 | 12:30479358:T:G | NA | NA | NA |
| rs2033867 | 2:175188281:G:A | NA | NA | NA |
| rs58691539 | 2:52526771:T:G | NA | NA | NA |
| rs7107977 | 11:915764:A:G | NA | NA | NA |

SNP, single-nucleotide polymorphism; Beta, the per allele effect on cannabis use; P value is for the genetic association. The included traits were found to be related with Cannabis-associated SNPs (P < 5×10-8). The results were obtained from PhenoScanner V2 website (http://www.phenoscanner.medschl.cam. ac.uk/).

Table S8. MR Analyses for the Association of Cannabis Use with Cardiovascular diseases

| Outcome | Method | OR (95%CI) | P value |
| --- | --- | --- | --- |
| CAD | Main IVW MR | 0.99 (0.94-1.04) | 0.61 |
|  | Weighted median Estimate | 0.97 (0.90-1.03) | 0.31 |
|  | MR-Egger Estimate | 0.91 (0.82-1.02) | 0.09 |
| MI | Main IVW MR | 0.97 (0.92-1.02) | 0.24 |
|  | Weighted median Estimate | 0.96 (0.89-1.04) | 0.29 |
|  | MR-Egger Estimate | 0.91 (0.81-1.03) | 0.12 |
| AS | Main IVW MR | 1.00 (0.95-1.06) | 0.96 |
|  | Weighted median Estimate | 0.97 (0.91-1.04) | 0.44 |
|  | MR-Egger Estimate | 1.07 (0.94-1.21) | 0.34 |
| AIS | Main IVW MR | 1.00 (0.94-1.06) | 0.92 |
|  | Weighted median Estimate | 0.97 (0.90-1.04) | 0.39 |
|  | MR-Egger Estimate | 1.01 (0.88-1.16) | 0.86 |
| LAS | Main IVW MR | 0.95 (0.83-1.09) | 0.48 |
|  | Weighted median Estimate | 0.93 (0.77-1.11) | 0.40 |
|  | MR-Egger Estimate | 0.94 (0.68-1.29) | 0.69 |
| CES | Main IVW MR | 0.98 (0.89-1.08) | 0.65 |
|  | Weighted median Estimate | 1.02 (0.89-1.16) | 0.80 |
|  | MR-Egger Estimate | 1.10 (0.88-1.38) | 0.38 |
| SVS | Main IVW MR | 1.09 (0.97-1.22) | 0.14 |
|  | Weighted median Estimate | 1.03 (0.88-1.21) | 0.73 |
|  | MR-Egger Estimate | 1.09 (0.84-1.42) | 0.50 |
| AF | Main IVW MR | 1.03 (0.99-1.07) | 0.20 |
|  | Weighted median Estimate | 1.05 (1.00-1.11) | 0.06 |
|  | MR-Egger Estimate | 1.06 (0.96-1.18) | 0.25 |
| HF | Main IVW MR | 1.00 (0.95-1.05) | 0.89 |
|  | Weighted median Estimate | 0.99 (0.93-1.06) | 0.81 |
|  | MR-Egger Estimate | 0.82 (0.80-1.00) | 0.05 |

CAD, coronary artery disease; MI, myocardial infarction; AS, any stroke; AIS, any ischemic stroke; LAS, large artery stroke; CES, cardioembolic stroke; SVS, small vessel stroke; P value is for the genetic association.

Table S9. Heterogeneity and directional pleiotropy analysis

| Outcome | Q | I^2^ | P-value for  heterogeneity | MR-Egger  intercept | P-value for  directional pleiotropy |
| --- | --- | --- | --- | --- | --- |
| CAD | 7.79 | 0 | 0.555 | 0.014 | 0.106 |
| MI | 5.83 | 0 | 0.768 | 0.011 | 0.250 |
| AS | 8.80 | 0 | 0.456 | -0.010 | 0.299 |
| AIS | 9.53 | 5.5% | 0.390 | -0.003 | 0.807 |
| LAS | 9.93 | 9.3% | 0.356 | 0.003 | 0.907 |
| CES | 4.41 | 0 | 0.883 | -0.022 | 0.232 |
| VSV | 7.24 | 0 | 0.612 | -0.001 | 0.976 |
| AF | 7.70 | 0 | 0.564 | -0.006 | 0.487 |
| HF | 11.53 | 21.9% | 0.241 | 0.018 | 0.036 |

CAD, coronary artery disease; MI, myocardial infarction; AS, any stroke; AIS, any ischemic stroke; LAS, large artery stroke; CES, cardioembolic stroke; SVS, small vessel stroke; AF, atrial fibrillation; HF, heart failure; P value is for the genetic association.

Table S10. Leave-one-out analysis for the association of Cannabis use with coronary artery disease and myocardial infarction

| SNP removed | CAD | | MI | |
| --- | --- | --- | --- | --- |
|  | OR(95% CI) | P value | OR(95% CI) | P value |
| rs35053471 | 0.99 (0.94-1.04) | 0.64 | 0.97 (0.92-1.02) | 0.27 |
| rs12518098 | 0.98 (0.93-1.03) | 0.39 | 0.97 (0.92-1.02) | 0.20 |
| rs4471463 | 0.99 (0.94-1.04) | 0.69 | 0.97 (0.92-1.02) | 0.25 |
| rs4984460 | 0.99 (0.94-1.04) | 0.55 | 0.97 (0.92-1.02) | 0.26 |
| rs7675351 | 0.98 (0.94-1.03) | 0.46 | 0.96 (0.91-1.01) | 0.13 |
| rs73067624 | 0.98 (0.93-1.03) | 0.42 | 0.97 (0.91-1.02) | 0.19 |
| rs2099149 | 0.98 (0.94-1.04) | 0.54 | 0.96 (0.91-1.02) | 0.18 |
| rs2033867 | 0.99 (0.95-1.04) | 0.76 | 0.98 (0.92-1.03) | 0.37 |
| rs58691539 | 1.00 (0.95-1.05) | 0.99 | 0.99 (0.93-1.04) | 0.60 |
| rs7107977 | 1.00 (0.94-1.06) | 0.99 | 0.97 (0.91-1.04) | 0.42 |

CAD, coronary artery disease; MI, myocardial infarction; P value is for the genetic association.

Table S11. Leave-one-out analysis for the association of Cannabis use with stroke

| SNP removed | AS | | AIS | |
| --- | --- | --- | --- | --- |
|  | OR(95% CI) | P value | OR(95% CI) | P value |
| rs35053471 | 1.00 (0.95-1.06) | 0.88 | 1.00 (0.95-1.05) | 0.93 |
| rs12518098 | 1.00 (0.95-1.05) | 0.99 | 0.99 (0.94-1.04) | 0.67 |
| rs4471463 | 1.01 (0.95-1.06) | 0.84 | 1.00 (0.95-1.06) | 0.97 |
| rs4984460 | 1.01 (0.96-1.06) | 0.72 | 1.01 (0.95-1.06) | 0.85 |
| rs7675351 | 1.00 (0.95-1.06) | 0.97 | 1.00 (0.95-1.06) | 0.98 |
| rs73067624 | 1.01 (0.96-1.06) | 0.82 | 1.00 (0.95-1.06) | 0.94 |
| rs2099149 | 1.00 (0.95-1.06) | 0.91 | 0.99 (0.94-1.05) | 0.82 |
| rs2033867 | 0.99 (0.94-1.05) | 0.75 | 0.99 (0.94-1.04) | 0.67 |
| rs58691539 | 0.98 (0.93-1.03) | 0.43 | 0.98 (0.93-1.03) | 0.46 |
| rs7107977 | 1.02 (0.96-1.08) | 0.54 | 1.02 (0.96-1.09) | 0.51 |

Table S11. Continued

| SNP removed | LAS | | CES | | SVS | |
| --- | --- | --- | --- | --- | --- | --- |
|  | OR(95% CI) | P value | OR(95% CI) | P value | OR(95% CI) | P value |
| rs35053471 | 0.95 (0.83-1.07) | 0.38 | 0.98 (0.88-1.08) | 0.63 | 1.10 (0.98-1.23) | 0.12 |
| rs12518098 | 0.95 (0.84-1.08) | 0.43 | 0.98 (0.89-1.09) | 0.74 | 1.09 (0.97-1.22) | 0.17 |
| rs4471463 | 0.95 (0.84-1.08) | 0.45 | 0.98 (0.88-1.08) | 0.65 | 1.12 (0.99-1.26) | 0.07 |
| rs4984460 | 0.96 (0.84-1.09) | 0.48 | 1.00 (0.90-1.10) | 0.94 | 1.09 (0.97-1.23) | 0.15 |
| rs7675351 | 0.99 (0.87-1.12) | 0.82 | 0.99 (0.89-1.09) | 0.79 | 1.07 (0.95-1.21) | 0.24 |
| rs73067624 | 0.94 (0.83-1.07) | 0.36 | 0.99 (0.89-1.09) | 0.76 | 1.09 (0.97-1.22) | 0.16 |
| rs2099149 | 0.94 (0.83-1.07) | 0.37 | 0.97 (0.88-1.08) | 0.56 | 1.07 (0.94-1.20) | 0.31 |
| rs2033867 | 0.94 (0.82-1.07) | 0.32 | 0.97 (0.87-1.07) | 0.52 | 1.07 (0.95-1.21) | 0.25 |
| rs58691539 | 0.93 (0.81-1.06) | 0.25 | 0.98 (0.88-1.08) | 0.65 | 1.10 (0.97-1.24) | 0.13 |
| rs7107977 | 1.03 (0.87-1.21) | 0.74 | 0.94 (0.83-1.07) | 0.38 | 1.15 (0.99-1.33) | 0.08 |

AS, any stroke; AIS, any ischemic stroke; LAS, large artery stroke; CES, cardioembolic stroke; SVS, small vessel stroke; P value is for the genetic association.

Table S12. Leave-one-out analysis for the association of Cannabis use with atrial fibrillation and heart failure

| SNP removed | AF | | HF | |
| --- | --- | --- | --- | --- |
|  | OR(95% CI) | P value | OR(95% CI) | P value |
| rs35053471 | 1.02 (0.98-1.06) | 0.27 | 0.99 (0.94-1.04) | 0.66 |
| rs12518098 | 1.03 (0.99-1.07) | 0.10 | 0.99 (0.94-1.03) | 0.56 |
| rs4471463 | 1.04 (1.00-1.08) | 0.05 | 0.99 (0.95-1.04) | 0.78 |
| rs4984460 | 1.03 (0.99-1.07) | 0.12 | 1.00 (0.95-1.05) | 0.99 |
| rs7675351 | 1.03 (0.99-1.07) | 0.21 | 1.00 (0.95-1.05) | 0.97 |
| rs73067624 | 1.03 (0.99-1.07) | 0.19 | 0.99 (0.95-1.04) | 0.71 |
| rs2099149 | 1.03 (0.99-1.07) | 0.19 | 0.99 (0.94-1.04) | 0.68 |
| rs2033867 | 1.04 (1.00-1.08) | 0.04 | 1.00 (0.95-1.05) | 0.86 |
| rs58691539 | 1.03 (0.99-1.07) | 0.16 | 1.01 (0.96-1.06) | 0.73 |
| rs7107977 | 1.01 (0.96-1.05) | 0.74 | 1.01 (0.96-1.07) | 0.65 |

AF, atrial fibrillation; HF, heart failure; P value is for the genetic association.
